# Differential cytokine expression by brain microglia/macrophages in primary culture after oxygen glucose deprivation and their protective effects on astrocytes during anoxia

**DOI:** 10.1186/s12987-015-0002-1

**Published:** 2015-02-28

**Authors:** Rawan Barakat, Zoran Redzic

**Affiliations:** Department of Physiology, Faculty of Medicine, Kuwait University, Mail: P O Box 24923, Safat, 13110 Kuwait

**Keywords:** Microglia, Microglia polarization, Astrocytes, Cytokines, Cerebral ischemia

## Abstract

**Background:**

Activation of microglia/macrophages following cerebral ischemia may be beneficial or detrimental for the survival of brain cells, an ambiguity in effects that has been explained by findings that ischemia can induce transformation of resting monocytes/macrophages into two different inflammation-related phenotypes, termed M1 and M2. The extent to which this differentiation depends on paracrine signaling from other brain cells is not clear. This study explored if oxygen glucose deprivation (OGD) can trigger expression of phenotype-specific markers in rat microglia/macrophages in primary culture, in absence/low abundance of other brain cells. Time pattern of these changes was assessed and compared to time-pattern that has been revealed *in vivo* previously. Effects of phenotype-specific cytokines on viability of astrocytes in primary culture during anoxia were also explored.

**Methods:**

Primary cultures of rat microglia/macrophages were exposed to 2h OGD and then incubated further under normal conditions; this was considered as a recovery period. Expression of mRNA for specific markers and secretion of phenotype-specific cytokines were explored at different time points by real time PCR and ELISA, respectively. Effects of cytokines that were secreted by microglia in primary culture after OGD on viability of astrocytes were determined.

**Results:**

Expression and secretion of M2 phenotype-specific markers and/or cytokines after OGD increased early after OGD and then decreased in the later stages of the recovery period. Expression and secretion of M1 phenotype-specific markers and cytokines did not show a common time pattern, but there was a tendency for an increase during the recovery period. All M1 phenotype-specific and two out of the three tested M2 phenotype-specific cytokines revealed protective effects on astrocytes during near-anoxia by a marked reduction of apoptosis.

**Conclusions:**

Time-pattern of expression/secretion of phenotype-specific markers suggested that polarization of the brain microglia/macrophages *in vitro* to M2 and M1 phenotypes were largely independent and likely dependent on signaling from other brain cells, respectively. Time-pattern of polarization to the M2 phenotype partially resembled time-pattern that has been seen *in vivo*. Effects of M1 phenotype-specific cytokines on primary culture of astrocytes were protective, thus largely opposite to effects that have been observed *in vivo*.

**Electronic supplementary material:**

The online version of this article (doi:10.1186/s12987-015-0002-1) contains supplementary material, which is available to authorized users.

## Background

Microglia/macrophages of the CNS account for ≈ 20% of brain glial population [1]. Under physiological conditions they exist in the resting state, which is characterized by a ramified phenotype [2], with cells continuously surveying the microenvironment with their processes [3-6]. Microglia/macrophages communicate with other cells of the neurovascular unit (NVU) *via* paracrine signaling mediated by cytokines and growth factors [1,7,8] and by cell-to-cell communication with neurons and astrocytes [3].

It has been postulated that activation of microglia and macrophages following brain injury aids survival of brain cells by removing cell debris [9,10], and by promoting neuronal sprouting and growth as observed *in vitro* [11]. However, experimental data suggest that microglia activation could also cause secondary expansion of infarction following cerebral ischemia that could worsen neurological outcome, an effect mediated by cytokines and chemokines released from activated microglia and impairing neurogenesis and axonal regeneration [12,13]. Although it is well known that there is a transient and significant increase in paracellular diffusion across the brain capillary endothelium following hypoxia/ischemia, it is not known to which extent microglia-derived cytokines contribute to these changes.

This ambiguity in the effects of microglia/macrophages activation has been partially elucidated by findings that peripheral monocytes and macrophages can transform upon stimulation into two different inflammation-related phenotypes, termed M1 and M2 [14] in a process being referred to as “polarization”. The M1 phenotype is characterized by a reduced ability for phagocytosis and by increased secretion of pro-inflammatory mediators; this phenotype occurs *in vivo* after prolonged stimulation of microglia/macrophages by inflammatory stimuli or could be induced *in vitro* as a response to stimulation by interferon (INF) -γ and pathogen-associated molecules, such as lipopolysaccharide (LPS) [15]. A characteristic phenotype of M1 cells includes up-regulation of CD16 Fc receptors, CD32, CD64, interleukin (IL) -1, IL-6, IL-12, IL-23, and tumor necrosis factor-alpha (TNFα). The M2 phenotype, on the other hand, has been induced *in vitro* by IL-4 [16]; cells with M2 phenotype exert phagocytic activity and produce anti-inflammatory cytokines IL-10 and transforming growth factor beta (TGF-β) and several growth factors, thereby promoting tissue remodeling and repair [17]. It has been shown recently that M2 microglia are in fact a mixed phenotype, consisting of cells that exhibit M2a or M2b phenotypes, the former one being characterized by increased expression of Arg1 and the latter one by increased expression mainly of IL-1RA and SOCS3 [18]. It has been also shown that only microglia expressing the M2a phenotype exerted neuroprotective effects during hypoxia/ischemia, while microglia of the M2b phenotype were injurious to neurons [18]. Astrocytes have been shown to act as endogenous sources of IL-4 in the brain [19], which suggests that microglia could be exposed to this polarizing cytokine *in vivo*. However, it is unclear whether the polarization of microglia/macrophages is mainly induced by paracrine signaling from neighboring cells or if it is induced by an internal (autocrine) mechanism after a specific stimulation and thus independent or only partially dependent on signaling from other brain cells.

A recent *in vivo* study used PCR and immunocytochemistry to explore polarization of microglia and macrophages after 1–14 days of reperfusion following 60 min of focal cerebral ischemia in mice [20]. The main finding was that there was an increase in microglia/macrophages with the M2 phenotype after 1–5 days of reperfusion, which was followed by a steady increase in microglia/macrophages with the M1 phenotype, so significantly more microglia/macrophages with the M1 phenotype were present after 10–14 days of reperfusion. This study found evidence that signaling from neurons was required for microglia/macrophage polarization towards the M1 phenotype and the authors hypothesized that signals from neighboring cells play a crucial role in microglia polarization.

Hypoxia/ischemia trigger up-regulation of hypoxia inducible factor (HIF) signaling in all cells; one of the HIF-downstream genes is STRA13, which in turn interferes with JAK/STAT pathway that is responsible for expression of various cytokines [21]. On the other hand, HIF2a partially controls expression of Interferon regulatory factor (Irf) 3 and possibly 4 and 5 [22]. These Irfs play a key role in phagocyte phenotype shift and polarization towards the M1 or towards the M2 phenotype [23-25]. Microglia is a target but also a source of IFNs α, β and γ [1,26]. However, it is not clear if these pathways play a role in microglia/macrophages polarization in the brain following ischemia.

The aim of this study was to determine if hypoxic/ischemic insult could be sufficient to trigger polarization of microglia *in vitro*, without interference of paracrine signaling from other brain cells. Results have revealed that OGD triggered a rapid change in gene expression and cytokine secretion in microglia in primary culture that partially resembled pattern of changes observed *in vivo*. Early after OGD the pattern of gene expression and cytokine secretion was similar to that found in the microglia/macrophages expressing the M2 phenotype. This initial change was followed by a mixed pattern of expression and secretion of cytokines that did not clearly resembled either of the two phenotypes. In addition to that, we explored effects of cytokines that were released from microglia after OGD on viability of astrocytes in primary culture during anoxia. This part of the study revealed unexpected results that all M1 phenotype-specific cytokines reduced cell apoptosis and increased viability of astrocytes.

## Methods

### Animals

Primary cultures were produced from 1–3 days old Sprague Dawley rats of both sexes, obtained from the Animal Resource Center (ARC) in the Faculty of Medicine (FOM), Kuwait University. Animal care and handling procedures complied with the standards of the International Council of Laboratory Animal Sciences and the guidance provided by ARC and has been approved by Ethic Committee of the FOM.

Primary cultures of astrocytes and microglia were produced according to established protocols [27,28]. After plating, cells were maintained in Dulbecco‘s Modified Eagle’s Medium supplemented with 10% (v/v) of fetal calf serum, vitamin C, antibiotic and antimycotic (all cell culture reagents and media from Gibco, Grand Island, USA). If not specified otherwise, the cell culture medium was changed every 2 days and primary cultures of microglia and astrocytes were used for experiments at days 7 and 10 after seeding, respectively. Primary cultures of microglia were either subjected to 2 h OGD that was in some cases followed by a recovery period or were used as controls.

### Oxygen and glucose deprivation protocol

Primary cultures were transferred to hypoxia glove box (Plas BY Labs, Lansing, MI, USA) with a gas mixture consisting of 5% CO_2_, 5% H_2_ in N_2_. The platinum catalyst was used to eliminate any remaining O_2_ and ≤0.3% of this gas was present during the course of OGD experiments. For these reasons the conditions applied in this study could be considered as near-anoxia. All buffers and media that were used for OGD experiments were left in the glove box for 12-24 h prior to experiments in order to equilibrate gas pressures. The temperature inside the box was maintained at 37 ± 1.5°C by an internally mounted heater. After being transferred to the glove box, primary cultures were washed with PBS and then incubated for 2 h in serum-, glucose- and pyruvate-free DMEM.

### Control conditions

The cultures were incubated in 5% CO_2_ in air at 37°C in serum-free DMEM (Gibco) that contained 5.5 mM glucose and 1.2 mM pyruvate for 2 h. Except for glucose and pyruvate, the composition of this DMEM was the same as of the DMEM used for the OGD protocol.

### Recovery

In some cases, when OGD or control protocols were completed, cell cultures were incubated in serum, pyruvate and glucose-containing DMEM in 5% CO_2_ in air at 37°C for a certain period of time. This was considered as a recovery protocol.

### Pharmacological induction of the M1 and M2 phenotypes

The M1 phenotype was induced in microglia *in vitro* by 48 h incubation in the medium containing 100 ng/ml of lipopolysaccharide (LPS) (Sigma, St. Louis, MI, USA) and 20 ng/ml of rat recombinant INFγ (R&D systems, Minneapolis, USA). The M2 phenotype was induced by 48 h incubation in the medium containing 20 ng/ml of rat recombinant IL-4 (Abcam, Cambridge, UK). At the end of these protocols the cell culture medium was collected and the cells were used for RNA extraction.

### Assessment of the amount of mRNA for specific markers of the M1 and M2 phenotypes

Total RNA was extracted from microglia in culture using NucleoSpin RNA XS kit (Marcherey Nagel, Düren, Germany), following manufacturer’s instructions, quantified using Epoch™ Microplate spectrophotometer (BioTek, Winooski, VT, USA) and stored at −70C. A total of 0.8 μg RNA was used for reverse transcription (RT), which was performed using Superscript II RNase H- Reverse transcriptase (Invitrogen, Waltham, MA, USA), random primers (Invitrogen) and deoxynucleotide triphosphates mix (RT positive samples). RT negative samples contained water instead of reverse transcriptase. Real-time PCR was performed using TaqMan probes at 7500 PCR cycler (Applied Biosystems, Waltham, MA, USA). 18 s rRNA was used as a housekeeping gene and this selection was based on findings that experimental procedures did not affect amount of this RNA in primary cultures. Expression of mRNA for the following six proteins was assessed: inducible nitric oxide synthase 2 (NOS2), low affinity immunoglobulin gamma Fc region receptor III (CD16), Fc fragment of IgG, low affinity IIb receptor (CD32) as M1 phenotype-specific markers and arginase 1 (Arg1), interleukin 10 (IL-10) and transforming growth factor beta 1 (TGFβ1) as M2 phenotype-specific markers. Relative gene expression was calculated as fold change in experimental versus corresponding control groups as described earlier [29]. Fold change values in cultures after 2 h OGD protocol were calculated against the amount of mRNA in cultures in control conditions for 2 h; fold change values in groups that were subjected to recovery protocols of various durations after 2 h OGD were calculated against the respective control groups that were subjected to the recovery protocol of the same duration. Since Ct values reflect exponential change in the amount of mRNA, the distribution of these values around means could not be considered as normal; for these reasons no statistical significance of the differences was obtained for these values.

### Enzyme-Linked Immunosorbent Assay (ELISA)

Concentration of cytokines that are typically secreted by activated microglia, M1 phenotype-specific cytokines TNFα, IL-12, IL-6 and M2 phenotype-specific cytokines TGFβ and IL-10 were assessed in the cell culture medium after different protocols using ELISA kits (Abcam) following manufacturers’ instructions and expressed as pg/mL.

### Determination of effects of cytokines released from activated microglia on viability of astrocytes during anoxia

Cytokines shown to be released by activated microglia were added to primary cultures of astrocytes to assess their potential protective or injurious effects during anoxia. Flasks were divided into two groups to be incubated in the presence and in the absence of cytokines, respectively. Each of the following cytokines were added to at least 8 flasks of the first group (all cytokines were from Abcam if not stated otherwise): M1 phenotype-specific cytokines - 200 pg/ml of TNFα, 500 pg/ml of IL-6 (Invitrogen), 400 pg/ml of IL-12 (Invitrogen) and M2 phenotype-specific cytokines - 275 pg/ml of TGFβ and 2200 pg/ml of IL-10 and 1000 pg/ml of IL-4, while an equal volume of the vehicle was added to at least 8 flasks from the second group. These concentrations were selected taking into account estimated average concentrations in control groups and at various time points after OGD protocol (i.e. an average that would be present in the medium) and also manufacturers’ recommendation on concentrations of cytokines that elicit biological effects. In the case of IL-4, the concentration was chosen based on the recommendation by the manufacturer. These two groups (with or without cytokines) were then subdivided into experimental groups and controls, with at least 4 flasks in each. Thus, four subgroups were analyzed for each cytokine:

- without cytokine, control conditions;
- with cytokine, control conditions;
- without cytokine, near-anoxia;
- with cytokine, near-anoxia.

Controls were incubated in 5% CO_2_ in air at 37°C for 24 h, while other flasks were incubated in 5% CO_2_, 5% H_2_ in N_2_ (near-anoxia) at 37°C for 24 h. After these protocols were completed, astrocytes were detached from flasks using TrypLE™ buffer (Gibco), and percentage of viable, apoptotic and necrotic cells in every sample was determined using Annexin V-R-PE kit (Southern Biotech, Birmingham, AL, USA) on a Cytomics FC 500 Flow cytometer (Beckman Coulter, Brea, CA, USA) according to manufacturer’s instructions.

### Statistics

All data are presented as mean ± SEM. A one-way or two-way analysis of variance was used to determine variable effects while a Tukey–Kramer post hoc test was used for pairwise comparisons. When only two groups were assessed, a Student’s t-test was employed. A *p* value of < 0 · 05 was considered statistically significant.

## Results and discussion

### Expression of mRNA for markers of polarized microglia after OGD and during the recovery period

The vast majority (>95%) of cells in primary culture of microglia were CD 11b positive (This is illustrated in Additional file 1). However, no differentiation could be made between the resident microglia and macrophages. Expression of mRNA for the six M1 and M2 phenotype-specific markers has been explored in these cultures. The obtained Ct values in control groups were (mean ± SEM) 17.4 ± 0.2, 22.9 ± 0.3, 33.1 ± 0.4, 32.8 ± 0.4, 31.9 ± 0.5, 24.3 ± 0.3 and 26.5 ± 0.4 for 18 s rRNA, TGFβ, IL-10, Arg1, NOS2, CD16 and CD32, respectively (n = 16-18). Since efficiency of the PCR reaction using TaqMan primers could be considered to be 2, it could be asumed that every PCR cycle doubled the amount of a specific sequence. Taking this fact into account it could be estimated that mRNA for TGFβ was only ≈ 45-fold less abundant than 18S rRNA; its abundance was closely followed by mRNA for CD16 and CD32. On the other hand, these cells contained ≈ 23,000, ≈43,000 and ≈ 52,000 fold less mRNAs for NOS2, Arg1 and IL-10 than 18S rRNA, respectively. Ct values for 18S rRNA remained within +/− 0.4 range in experimental and control groups, which indicated that its amount was not affected by OGD protocol or recovery. Ct values for all six genes of interest were within a narrow range in all control groups for up to 10 days of the recovery protocol, which indicated that these mRNA species were stabily expressed and levels were not affected by the duration of primary cultures for the specified period of time. However, there was a decline in the amount of both 18 s rRNA and mRNAs for most genes of interest if the recovery period was longer than 10 days (data not shown); for this reason, this study was limited to a recovery period 1–10 days.

**Figure 1:**
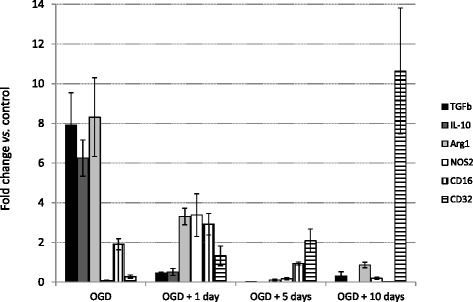
**The effect of oxygen glucose deprivation and recovery on expression of mRNA for six M1 and M2 phenotype-specific markers.** Each bar represents fold change in gene expression in OGD group that was calculated against the amount of mRNA in corresponding control group. M2 specific: TGFβ, IL-10, Arg1, M1 specific: NOS2, CD16, CD32. Data are presented as mean ± SEM from 5–8 separate RNA samples.

Next, we explored amount of these mRNAs after 2 h OGD protocol and after 1, 5 and 10 days recovery period that followed OGD. The results are presented in Figure 1. The duration used in the OGD protocol was based on previous findings that during 2 h OGD cell death of microglia remained low, below 5%, while it increased significantly after 6 h OGD [30], on a therapeutic goal to establish reperfusion after stroke within first 1-2 h after the onset of symptoms and on a fact that majority of *in vivo* studies used middle cerebral artery occlusion for the duration of 30 min-2 h.

A common pattern of changes in the mRNA for the three markers of the M2 phenotype was revealed, with initial increase after OGD treatment, followed by a decrease during the recovery period (Figure 1). There were 7.9, 6.2 and 8.3 fold changes after 2 h OGD in mRNA for TGFβ, IL-10 and arginase 1, respectively, while after 1 day recovery these three mRNAs were 0.44, 0.51 and 3.3 fold to that in control groups and were further reduced after 5 and 10 days of recovery. However, no common pattern in mRNA expression changes could be established for the three markers of the M1 phenotype. OGD treatment caused initial decrease in the mRNAs for CD32 and NOS2 and a marginal increase in the mRNA for CD16; this was followed by an increase in the mRNAs for all three markers after 1-day recovery. There was a further steady increase in mRNA for CD-32 during the recovery period, reaching a ≈ 10 fold increase after 10 days, while the mRNAs for CD16 and NOS2 decreased, reaching 0.02 and 0.2 fold changes after 10 days recovery. A previous *in vivo* study has shown that after 60 min occlusion of the middle cerebral artery in mice there was a biphasic change in expression of mRNA for eight markers of the M1 and M2 phenotypes, with mRNA for markers of the M2 phenotype increasing earlier during recovery period (3–5 days), and mRNA for markers of the M1 phenotype increasing steadily during the recovery period for up to 14 days [20]. Based on these data, it was concluded in that study that cerebral ischemia triggered expression of mRNA encoding markers of the M2 phenotype in the first 3–5 days following ischemia, which was accompanied by a steady increase in mRNA encoding markers of the M1 phenotype during the recovery period. Thus, patterns of mRNA expression changes that were revealed in the present study partially resembled those revealed *in vivo*.

A different pattern of expression of mRNAs was reported after traumatic brain injury in Wistar rats, with both M1 and M2 phenotype – associated markers reaching peak values 5 days after the injury [31]. This indicates that pattern of polarization of microglia is not fully “preprogrammed” and that this pattern changes according to the initial stimuli that trigger the responses.

### Release of cytokines by activated microglia after pharmacological induction of the M1 or M2 phenotype

In this part of the study we first induced polarization of microglia in culture towards the M1 or the M2 phenotype by 48 h incubation in the cell culture medium containing specific cytokines. Both of these protocols induced a quick (within 2-3 h) retraction of cell processes; cells became oval and then apparently smaller than resting microglia/macrophages (data not shown). Similar changes in their phenotype were observed after 2 h OGD. Incubation with LPS/IFNγ caused 3, 6 and 12-fold increases in concentration of IL-6, IL-12 and TNFα, respectively (Table 1), while concentrations of the two cytokines that are specific for the M2 phenotype, TGFβ and IL-10, were reduced to 62% and 56% of those in control. This indicated that incubation with LPS/IFNγ caused differentiation of resting microglia/macrophages towards the M1 phenotype. No clear changes could be established after 48 h incubation of microglia with IL-4, a protocol that was supposed to trigger differentiation towards the M2 phenotype. There was a significant, 3.3 and 2.6-fold increase in concentration of the M2 phenotype-specific cytokines TGFβ1 and IL-10 in the medium, respectively. However, concentrations of the two M1 phenotype -specific cytokines, IL-6 and TNFα, has also increased significantly to 147% and 220% of their values in controls (*p* < 0.05 and *p* < 0.01, respectively), while concentrations of IL-12 did not change significantly (*p* > 0.05).

**Table 1:** **Concentrations of cytokines in the medium (pg/ml) after 48 h incubation of microglia with specific microglial activators (LPS/INFγ and IL-4) and in control groups**

	**Control**	**LPS/INFγ**	**IL-4**
TNFα	47.2 ± 4.0	567.8 ± 71.2**	107.5 ± 5.4*
IL-12	111.3 ± 32.7	632 ± 89.8**	79.5 ± 6.9
IL-6	715.9 ± 33.7	2262.7 ± 115.8**	1042.5 ± 42.6**
TGFβ	458.9 ± 21.9	286 ± 8.7**	1536.4 ± 116.4**
IL-10	1520.2 ± 48.0	852.4 ± 21.8**	3970.2 ± 277.9**

All data are presented as mean ± SEM from 4 different samples. Signs mean: * = *p* < 0.05 *versus* control; ** = *p* < 0.01 *versus* control.

### Release of cytokines from microglia during OGD protocol and recovery period

**Figure 2:**
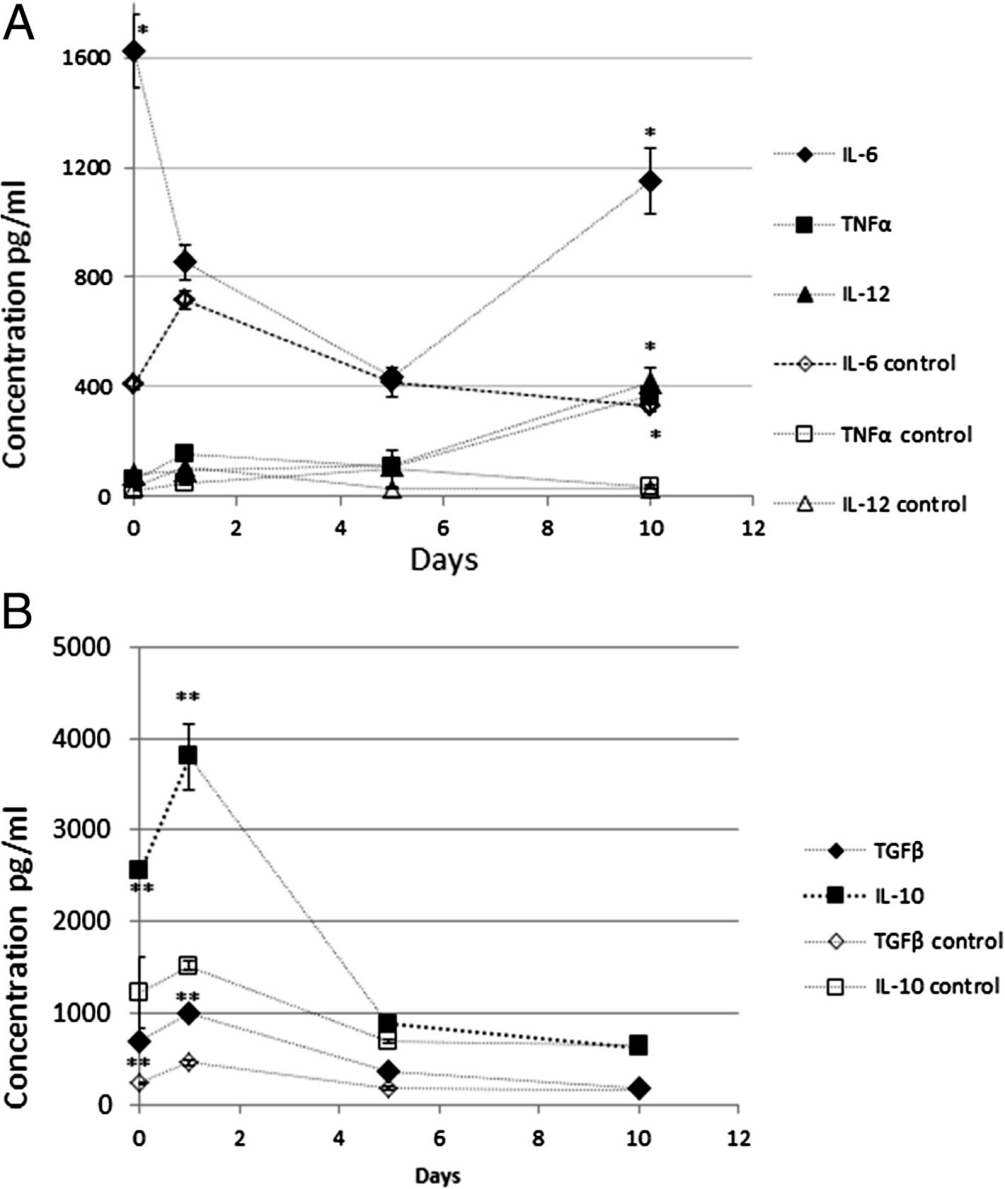
**Concentration of the M1-specific (A) and M2-specific (B) cytokines in cell culture media collected from control groups and from cultures that were exposed to OGD protocol.** As it could be seen from the figure, there was a common pattern of concentration changes after OGD protocol for the M2-specific cytokines **(panel B)**, with their concentration in the medium increasing immediately after the OGD protocol and then decreasing during the recovery period. The opposite pattern was revealed for the two M1-specific cytokines, TNFa and IL-12, with their concentrations being low after OGD protocol and then increasing during the recovery period, reaching the highest values after 10 days **(panel A)**. A pattern for the M1-specific cytokine IL-6 was different, with an initial increase in concentration after the OGD protocol, then decreasing during the recovery period and increasing again between days 5–10. Data are presented as mean ± SEM from 4 samples that were taken from 4 different flasks, * = *p* < 0.05, ** = *P* < 0.01, cultures exposed to OGD versus control.

Time-patterns of cytokine release from microglia after 2 h OGD and during the recovery period were also assessed (Figure 2). One-way ANOVA revealed a significant effect of the recovery period on concentrations of all 5 cytokines in the medium (*p* < 0.001); no significant effect of the time of incubation on the concentration of these cytokines was observed in the control groups (*p* > 0.05).

A common time-pattern of concentration change during recovery was revealed for the two M2 specific cytokines, TGFβ1 and IL-10 (Figure 2B): concentrations in the medium at day zero were significantly higher (*p* < 0.01 for IL-10, and *p* < 0.001 for TGFβ) than in the medium from controls, which suggested that microglia release these cytokines during OGD. This was followed by a further increase in concentrations in the medium from cultures that were exposed to OGD between days 0 and 1, and then by a large decrease in concentrations between days 1 to 10, so at day 10 concentrations of both cytokines in OGD groups did not differ significantly from control groups (*p* > 0.05).

No common time-pattern was revealed for all M1 phenotype-specific cytokines: concentrations of IL-12 and TNFα in the cell culture media at days 0, 1 and 5 were relatively low and did not differ significantly from those in control groups (*p* > 0.05); this was followed by a 4-fold increase in concentrations between days 5 and 10, so concentrations in the medium at day 10 were significantly higher (*p <* 0.001) when compared to concentration in control group at day 10 or to concentrations in OGD groups at any other time point (Figure 2A). Concentration of IL-6 in the medium was significantly higher than in control group at day 0, (*p* < 0.001), which indicated that microglia released this cytokine when exposed to OGD, but was much lower at days 1 and 5 (*p* > 0.05 vs. control) and then, similarly to concentrations of IL-12 and TNFα, increased several fold between days 5 and 10 (Figure 2A).

These data, together with PCR results, demonstrated that microglia *in vitro* (as a monoculture) exhibited some features of the M2 phenotype following OGD; however, these features changed during the time course of the recovery protocol. This finding suggests that paracrine signaling from other brain cells, which were to large extent absent from primary cultures, was not essential to cause changes in gene expression and cytokine secretion that was consistent with polarization into the M2 phenotype. However, as primary cultures were not absolutely pure, effects of paracrine signaling from small number of contaminant cells (mainly astrocytes) on expression and secretion in microglia/macrophages could not be entirely excluded. Previous reports concluded that microenvironment played a crucial role in microglia polarization [11], especially with respect to induction of the M1 phenotype or in the “shift” between the M1 and M2 phenotypes [11]. However, the mechanisms that trigger polarization remain unclear. In macrophages interferon- response factors play a role in phagocyte phenotype shift, with interferon regulatory factors (IRF) 4 and 3 encoding a key transcription factor that controls M2 macrophage polarization [23,24] , while IRF 5 encoded a transcription factor that controls M1 polarization [25]. Other studies have shown involvement of Notch signaling and CCAAT-enhancer-binding proteins’ cascade [32] in induction of polarization, and shift between the M2 and M1 phenotypes.

### Effects of microglia-derived cytokines on viability of astrocytes during anoxia

It has been shown *in vivo* that microglia exert protective effects on neurons after focal cerebral ischemia [33]. However, an *in vitro* study revealed that these protective effects were mediated either by resting microglia or by polarized microglia with the M2 phenotype, while polarized microglia with the M1 phenotype exerted injurious effects on neurons in culture [11]. It has also been reported that microglia-conditioned medium was protective for astrocytes that were exposed to OGD; these effects were primarily mediated by glial cell line-derived neurotrophic factor (GDNF) -related expression of extracellular signal-regulated kinase and nuclear factor-kappa B in astrocytes [34,35]. Expression and secretion of GDNF by activated microglia has been well documented [36]. A previous *in vitro* study has also shown that microglia conditioned medium was protective for astrocytes and brain endothelial cells in primary culture during OGD and that this effect was, at least partially, mediated by IL-6 and TGFβ [30].

In this study we found that activated microglia secreted at least five different cytokines. Therefore, we explored effects of these cytokines on astrocytes exposed to near-anoxia or control conditions. However, it should be noted that in these *in vitro* experiments microglia/macrophages released cytokines into a large volume of cell culture medium, while if this process occurs at a similar rate *in vivo*, cytokines will be secreted into the extracellular space that has smaller volume; this might produce higher concentrations *in vivo* from those observed *in vitro*. For each sample percentages of cells that were viable, early apoptotic, late apoptotic and necrotic were calculated (Illustrated in Additional file 2). The results obtained were surprising. A common effect by all cytokines tested was a reduction in either early (IL-12, IL-6) or in late (IL-10) or in both types of apoptosis (TNFα, IL-4, TGFβ). This reduction was in some cases marginal (IL-10) and in some cases substantial (TNFα, IL-12, TGFβ and IL-4). The second common pattern was that all cytokines, except IL-12 and TGFβ, caused a marginal but significant increase in necrosis of astrocytes during anoxia.

**Figure 3:**
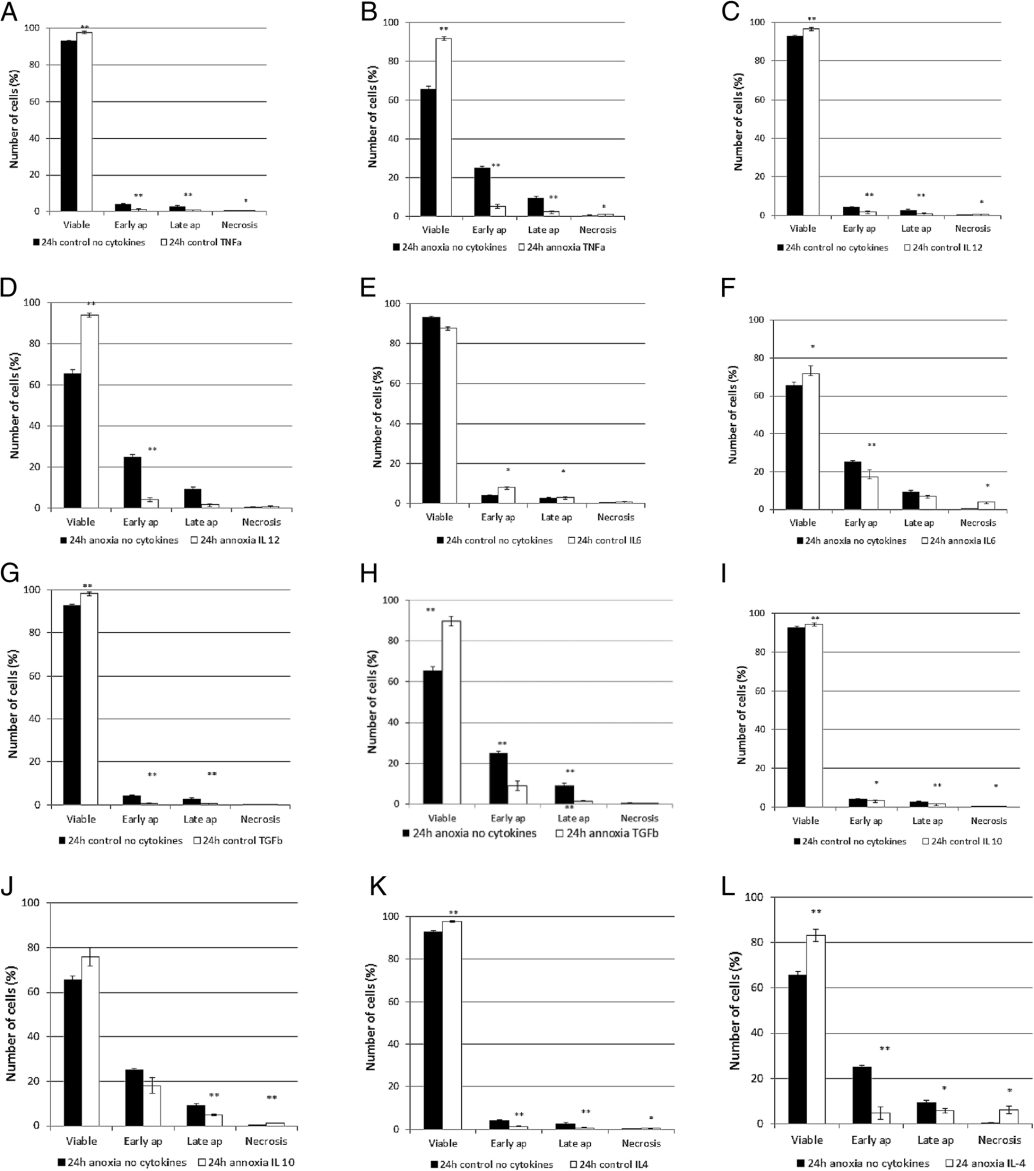
**Viability of astrocytes in culture after exposure to 24 h anoxia.** Panels **B, D, F, H, J, L** represent the data obtained in the presence or in the absence of cytokines during anoxia, while panels **A, C, E, G, I,** and **K** represent the data obtained in the corresponding control groups. The M1 phenotype-specific cytokines (TNFα, IL-12, IL-6) and M2 phenotype-specific cytokines (TGFβ and IL-4), but not IL-10, exerted protective effects on astrocytes during anoxia. Data are presented as mean ± SEM from 4–6 separate primary cultures of astrocytes, * = *p* < 0.05, ** = *P* < 0.01 with cytokines versus control.

The third common pattern was that all three cytokines that were reported to be M1 phenotype specific (and thus expected to reveal injurious effects on other brain cells) demonstrated protective effects. TNFα exerted a marginal but highly significant increase in astrocytes’ viability under control conditions when compared to viability in the absence of this cytokine (*p* < 0.001) (Figure 3a). This was accompanied by a small but significant decrease in the percentage of cells in early and late apoptosis (*p* < 0.01) and a marginal but significant increase in necrosis (*p* < 0.05) (Figure 3a). A similar pattern was also observed when astrocytes were exposed to 24 h anoxia in the presence of TNFα; viability of astrocytes was >90% when TNFα was present, which was a large increase when compared to viability of ≈ 65% during anoxia when TNFα was not added to the medium (*p* < 0.001) (Figure 3b).This was accompanied by a large decrease in percentage of cells that were in early and late apoptosis (*p* < 0.01) and by a marginal, but significant increase in necrosis (*p* < 0.05). A very similar pattern was demonstrated by IL-12 (Figure 3c and d) and IL-6 (Figure 3e and f).

Surprisingly, IL-10 reported to be M2 phenotype-specific (thus, expected to exert protective effects on astrocytes) did not affect viability of cells significantly (Figure 3I, J). It caused a minor reduction in apoptosis, but also a marginal increase in necrosis, so the viability of astrocytes did not change in the presence of this cytokine. However, in the presence of TGFβ and IL-4 a large reduction in apoptosis was revealed, so viability of astrocytes increased significantly (Figure 3G, H, K, L).

It may be hypothesized that a possible explanation for the discrepancies between these *in vitro* findings and findings *in vivo* could be that effects of these cytokines on astrocytes *in vivo* were largely mediated indirectly, through their effects on other neighboring cells.

The effects of astrocytes on induction and maintenance of a specific phenotype of brain capillary endothelial cells are well-known and have been reviewed extensively [37]. Although other cells of the NVU could affect the blood brain barrier, astrocytes play a pivotal role in maintaining its integrity [37,38]. For these reasons, the observed protective effects of microglia-derived cytokines on astrocyte viability during anoxia indicates that *in vivo* they could be protective for the blood brain barrier during hypoxia/ischemia.

## Conclusions

Exposure of microglia/macrophages in primary culture to OGD protocol triggered changes in mRNA expression for several markers of the M1 and M2 phenotypes and affected the secretion of the M1 and M2-specific cytokines. These findings suggest that paracrine signaling from other brain cells may not be essential for microglia polarization after cerebral ischemia. Based on the pattern of mRNA changes and on the release pattern of the phenotype-specific cytokines, it appears that OGD triggered polarization towards the M2 phenotype, which was followed during recovery by patterns that suggested presence of cells showing both phenotypes. Surprisingly, all three M1 phenotype-specific cytokines exerted protective effects on astrocytes in primary culture during anoxia. Although these *in vitro* data may not accurately reflect *in vivo* conditions, they could provide new insights into events that occur during and after cerebral ischemia.

## Additional files

Additional file 1:
**Purity of microglia/macrophages in primary culture.** A 5-day old primary culture of brain microglia/macrophages stained with mouse monoclonal anti-CD11b antibody (Abcam) and then with goat anti-mouse IgG antibody that is conjugated to fluorescein isothiocyanate. Mounting medium contained 4′ ,6-diamidino-2-phenylindole (DAPI) for nuclear staining. CD11b is expressed on the surface of many leukocytes including monocytes and macrophages, but not by astrocytes and neurons. As seen in this image, the vast majority of cells in the culture were CD11b-positive. Additional file 2:
**Estimation of cell viability, apoptosis and necrosis in astrocytes.** Primary astrocytes in control conditions (A) or after 24 h anoxia (B) were stained with Annexin V – Phycoerythrin (PE) and 7-Amino-actinomycin (7-AAD). Cells located in the B4 quadrant were early, apoptotic, 7-AAD-negative/Annexin PE-positive. Cells in the B3 quadrant were viable, 7-AAD-negative/Annexin PE-negative. Cells in the upper quadrants B1 (7-AAD-positive/annexin PE-negative) and B2 (7AAD-positive/annexin PE-positive) were necrotic and late apoptotic, respectively. A representative panel from each group is shown.

## Abbreviations

OGD: oxygen glucose deprivation
TNFα: tumor necrosis factor alpha
TGFβ: transforming growth factor beta
HIF: hypoxia inducible factor
JAK/STAT: Janus kinase/Signal Transducer and Activator of Transcription
pVHL: Von Hippel–Lindau tumor suppressor
Irf: Interferon regulatory factor
LPS: lipopolysaccharide
NOS: nitric oxide synthase
Arg: Arginase
GDNF: glial cell line-derived neurotrophic factor
