# Supplementary figures and images for: Differential cytokine expression by brain microglia/macrophages in primary culture after oxygen glucose deprivation and their protective effects on astrocytes during anoxia
Source: Fluids Barriers CNS. 2015 Feb 28;12:6. doi: 10.1186/s12987-015-0002-1 (PMC4392752; doi:10.1186/s12987-015-0002-1)

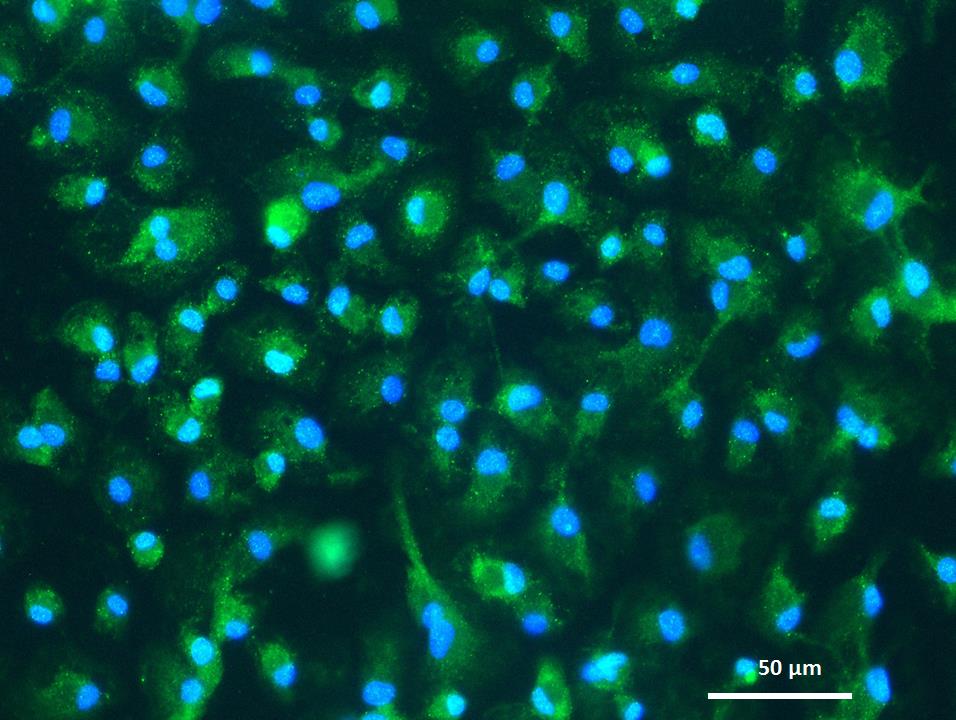

Supplement: Additional file 1: — Purity of microglia/macrophages in primary culture. A 5-day old primary culture of brain microglia/macrophages stained with mouse monoclonal anti-CD11b antibody (Abcam) and then with goat anti-mouse IgG antibody that is conjugated to fluorescein isothiocyanate. Mounting medium contained 4′ ,6-diamidino-2-phenylindole (DAPI) for nuclear staining. CD11b is expressed on the surface of many leukocytes including monocytes and macrophages, but not by astrocytes and neurons. As seen in this image, the vast majority of cells in the culture were CD11b-positive. [file 12987_2015_2_MOESM1_ESM.jpeg]

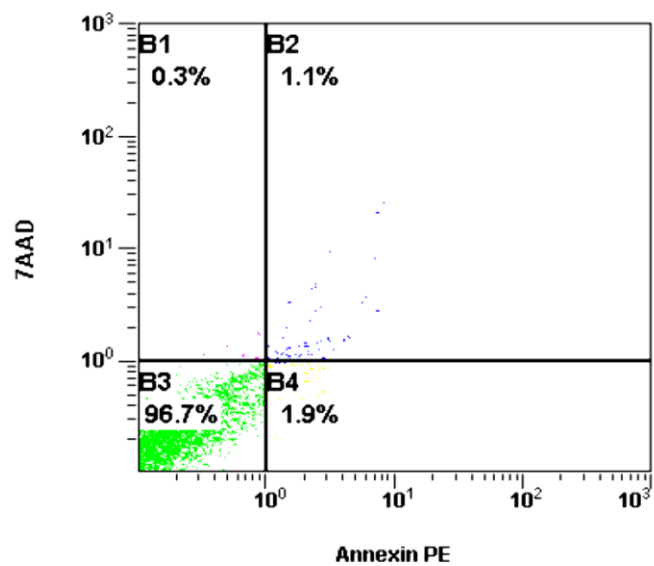

A

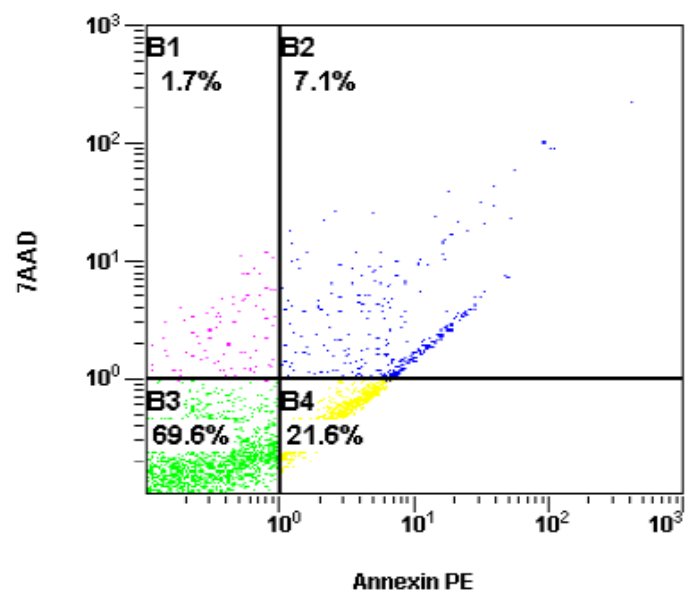

B

Supplement: Additional file 2: — Estimation of cell viability, apoptosis and necrosis in astrocytes. Primary astrocytes in control conditions (A) or after 24 h anoxia (B) were stained with Annexin V – Phycoerythrin (PE) and 7-Amino-actinomycin (7-AAD). Cells located in the B4 quadrant were early, apoptotic, 7-AAD-negative/Annexin PE-positive. Cells in the B3 quadrant were viable, 7-AAD-negative/Annexin PE-negative. Cells in the upper quadrants B1 (7-AAD-positive/annexin PE-negative) and B2 (7AAD-positive/annexin PE-positive) were necrotic and late apoptotic, respectively. A representative panel from each group is shown. [file 12987_2015_2_MOESM2_ESM.pdf]
